# How robust are egocentric and altercentric interference effects in social cognition? a test with explicit and implicit versions of a continuous false belief task

**DOI:** 10.3389/fpsyg.2023.1142302

**Published:** 2023-07-10

**Authors:** Feride Nur Haskaraca, Marina Proft, Ulf Liszkowski, Hannes Rakoczy

**Affiliations:** ^1^Department of Developmental Psychology, University of Göttingen, Göttingen, Germany; ^2^Department of Developmental Psychology, University of Hamburg, Hamburg, Germany

**Keywords:** social cognition, egocentric bias, altercentric bias, Sandbox task, mouse tracking

## Abstract

It has been long assumed that meta-representational theory of mind (ToM) -our ability to ascribe mental states to ourselves and other people- emerges around age four as indicated in performance on explicit verbal false belief tasks. In contrast, newer studies assessing false belief understanding with implicit, non-verbal measures suggest that some form of ToM may be present even in infancy. But these studies now face replication issues, and it remains unclear whether they can provide robust evidence for implicit ToM. One line of research on implicit ToM, however, may remain promising: Studies that tap so-called altercentric biases. Such biases occur when agents in their judgments about the world are influenced (perform slower, more error-prone) in light of another agent’s deviating perspective even if that perspective is completely irrelevant to the task; they thus can be seen as indicators of spontaneous and implicit ToM. Altercentric biases are the mirror images of egocentric biases (agents are influenced by their own perspective when evaluating another agent’s deviating perspective). In three studies with adults, we aimed to tap both egocentric and altercentric interference effects within the same task format. We used the so-called Sandbox task, a false belief task with continuous locations. In Study 1, we tested an online adaptation of the Sandbox task, which we also used to explore potential cross-cultural differences in these biases. Studies 2 and 3 combined the Sandbox task with mouse-tracking measures. These studies revealed neither egocentric nor altercentric biases. These null results are discussed with regard to the question whether absence of evidence here may present evidence of absence of such spontaneous perspective-taking biases or merely false negatives.

## 1. Introduction

Theory of Mind (ToM), the ability to ascribe mental states to ourselves and other people, is central to our nature as social beings. At the heart of ToM is the capacity to represent people’s representational states, i.e., meta-representation. The clearest indicator of this capacity is representing how others represent the world and act accordingly, even when their representation is inaccurate (hence a misrepresentation). The litmus tests for the development of meta-representational ToM have been False Belief (FB) tasks. In the most widely used so-called change-of-location FB task, participants hear a story in which an object is re-located either in the absence (FB condition) or the presence (True Belief control condition) of a protagonist. Then the participants are asked where the protagonist will look for the object (e.g., Wimmer and Perner, 1983). Decades of research using the change-of-location FB task, or other FB tasks such as unexpected contents (e.g., Perner et al., 1987), collectively demonstrated that children come to solve these tasks around 4 years of age (Wellman et al., 2001). Furthermore, children showed similar performance shifts around age four in superficially different ToM tasks, indicating a common cognitive capacity underlying these tasks (e.g., Perner and Roessler, 2012). This body of evidence was the basis for assuming that meta-representational ToM emerges in the preschool years, with the fundamental conceptual transition around age four.

However, newer findings in the last two decades have challenged this assumption. Implicit FB tasks infer FB understanding from participants’ spontaneous looking behavior or behavioral interactions -rather than explicit verbal answers. They include violation of expectation (VoE), anticipatory looking (AL), and interactive tasks. VoE studies show that children display longer looking times when an agent acts inconsistently with her (false) belief (Onishi and Baillargeon, 2005). AL paradigms reveal that, when presented with an FB scenario, children anticipate the agent to behave in accordance with her (false) belief, as indicated by their looking patterns (Southgate et al., 2007). Finally, findings from interactive tasks suggest that children can take their partners’ (false) beliefs into account when responding to them in communicative and other interactions (Buttelmann et al., 2009; Knudsen and Liszkowski, 2012). These findings suggest that some form of implicit sensitivity to others’ beliefs is present earlier than age four, perhaps even in the first year of life (see Scott and Baillargeon, 2017 for a review). Furthermore, a converging line of research with adults suggests that these implicit (and largely automatic) capacities remain intact over the lifespan (e.g., Kovács et al., 2010; Samson et al., 2010; Schneider et al., 2012). Far-reaching theoretical accounts build on these seminal findings, in particular nativist theories (Leslie, 2005; Scott and Baillargeon, 2017) or two-systems approaches (Apperly and Butterfill, 2009; Low et al., 2016).

According to nativist accounts, the findings from the implicit FB tasks suggest that ToM is a domain-specific capacity that is in place early in ontogeny or maybe even innate. Nativist accounts claim that explicit verbal FB tasks cannot uncover these early competencies because of performance factors, such as linguistic and inhibitory demands of the standard FB tasks (e.g., Leslie, 2005; Scott and Baillargeon, 2017). According to two-system accounts, the findings of the implicit FB tasks could tap an early-developing mind-reading system. This system, in contrast to the full-fledged flexible meta-representational system that develops in a more protracted form, is evolutionarily ancient, emerges early in ontogeny, and operates efficiently and broadly automatically, but is crucially limited in its (meta-) representational powers (Apperly and Butterfill, 2009; Low et al., 2016).

However, the empirical basis for the implicit ToM understanding is not solid yet. To date, only a relatively small number of studies provided positive findings for implicit ToM measures. Most of these studies were conducted by relatively few labs, and many had small sample sizes (= < 10 per condition in Onishi and Baillargeon, 2005; Southgate et al., 2007; Senju et al., 2010; or = < 25 per condition in Buttelmann et al., 2009). Moreover, the subsequent replication attempts with infants, children, and adults have produced mixed findings and raised doubts about the replicability and validity of the existing implicit ToM measures (for reviews, see Kulke et al., 2018a; Barone et al., 2019; Rakoczy, 2022). One can identify at least three issues regarding the standard implicit measures of ToM. First, all measures (i.e., VoE, AL, and interaction paradigms) encountered failed replication attempts in the last years (e.g., Yott and Poulin-Dubois, 2016; Burnside et al., 2018; Dörrenberg et al., 2018; Poulin-Dubois and Yott, 2018; Powell et al., 2018; Priewasser et al., 2018; Schuwerk et al., 2018). These replication attempts ranged from conceptual to direct, or even self-replications (e.g., Kampis et al., 2021), which makes it hard to attribute the failed replications to poor implementations of the original procedures. Second, all types of implicit ToM measures have shown poor construct validity so far: the earlier positive findings were replicable in some of the later replication attempts only under certain conditions with confounds (e.g., imbalanced number of cues for one answer). When these confounds were eliminated by introducing appropriate controls, the effects disappeared and the measure in question became non-replicable (e.g., Low and Watts, 2013; Powell et al., 2018; Priewasser et al., 2018; Kulke et al., 2018b). Lastly, implicit tasks have been shown to lack convergent validity. Several recent studies found minimal or no systematic correlations between the three standard measures of implicit ToM, nor even with the different tasks of the same type, which supposedly tap the same ability (e.g., Yott and Poulin-Dubois, 2016; Dörrenberg et al., 2018; Poulin-Dubois and Yott, 2018; Powell et al., 2018; Kulke et al., 2018a,b). These issues point to serious reliability and validity issues regarding the standard implicit measures, i.e., VoE, AL, and interaction paradigms. Thus, whether implicit measures reveal robust evidence for ToM in infancy and automatic and implicit forms of ToM throughout the lifespan remains unclear. Additionally, it remains unclear how early implicit and later explicit ToM performance may be related developmentally, with some studies speaking for continuity (Sodian et al., 2016, 2020) why others fail to replicate longitudinal continuity patterns (Poulin-Dubois et al., 2023; for recent debate see and Sodian, 2023 and Poulin-Dubois et al., 2020).

However, in the last decade, another phenomenon has come into focus as a potentially promising indicator of implicit perspective-taking: Altercentric interference or altercentric bias effects suggest that our own judgments or behaviors are influenced by how other people perceive the world, indicating that we implicitly represent their beliefs and perspectives - even when those are entirely irrelevant or interfere with our own task (Kampis and Southgate, 2020; Southgate, 2020). For example, across different studies, participants were found to be slower and more error-prone in counting objects if another agent was present in the scene but had an incongruent perspective on the object (e.g., only saw a subset of objects; Kovács et al., 2010; Samson et al., 2010). Interestingly, the interference seems to occur spontaneously and automatically, without subjects consciously or intentionally focusing on others’ perspectives. Theoretically, this bias could thus reflect more unambiguously implicit ToM processes than the standard implicit FB tasks (i.e., VoL, AL, or interactive tasks). And from a methodological perspective, altercentric bias tasks have several potential advantages over typical implicit FB tasks. For instance, they can provide more fine-grained, continuous measures of implicit ToM (participants can be more or less subject to altercentric interference).

In addition, altercentric bias measures are particularly interesting and promising from a methodological point of view: they allow researchers to construct structurally analogous tasks to tap implicit and explicit ToM within one task format such that the two types of tasks differ merely with regard to the critical test question. On the one hand, in implicit versions employing altercentric bias, participants are asked to make a factual judgment about the world (e.g., How many dots are there? / Where is an object?) in the presence of an irrelevant agent who does or does not share their perspective. If participants are slower or more error-prone in their own factual judgments when the other agent has a deviant perspective, this indicates altercentric bias. On the other hand, the explicit versions exploit the so-called egocentric bias, which refers to the influences of one’s own knowledge when judging others’ perspectives. In the explicit versions, participants are asked about the other agent’s perspective or behavior (e.g., How many dots does the agent see? / Where will the agent look for the object?). If participants become slower or more error-prone in these perspective judgments when their own perspective is different from the agents’, this indicates egocentric bias. This bias could then be used to infer the explicit ToM ability of participants: more interference from one’s own perspective -even if the task asks to take other’s perspective- means poorer ToM.

So far, these two biases have been implemented together in the so-called Dot Perspective Task (Samson et al., 2010). In this task, adult participants were asked to judge the number of dots presented in a scene either from their own perspective (SELF condition) or as seen by an on-screen avatar (OTHER condition). Each condition featured two types of trials: consistent versus inconsistent. In consistent trials, all dots were equally visible for the participant and the avatar, and their perspectives were thus consistent. In inconsistent trials, some of the dots visible for the participant were behind the avatar, therefore, not visible to it. The two perspectives were thus inconsistent. This study revealed that participants were slower and made more errors when detecting the number of dots in inconsistent trials compared to consistent trials. These results were interpreted as providing evidence for both altercentric and egocentric interference effects in SELF and OTHER conditions, respectively.

The dot perspective task has been one of the few measures in which both biases are obtained using the same task format. However, this task is not free from replication issues and validity debates. Some of the later studies using the variations of the dot-perspective task either revealed no bias (e.g., Conway et al., 2017) or the biases were subject to alternative explanations by domain-general mechanisms rather than implicit mentalizing (e.g., Santiesteban et al., 2014; Cole et al., 2016; Conway et al., 2017; O’Grady et al., 2017). As an example of the latter, Santiesteban et al. (2014) tested participants in two different versions of the dot perspective task: some trials featured an avatar as in previous experiments, and in the other trials, the avatar was replaced by an arrow with similar low-level features such as color, size, and orientation. They found comparable altercentric interference effects in avatar and arrow conditions, suggesting that the so-called altercentric bias effects may reflect general cognitive processes such as spatial cueing rather than specifically social-cognitive processes of perspective-taking (but see Michael et al., 2018).

The rationale of the present study is thus to construct alternative tasks to tap implicit and explicit perspective-taking abilities through altercentric and egocentric biases, respectively. To this end, we are capitalizing on an established explicit continuous FB task, the so-called Sandbox task (e.g., Sommerville et al., 2013). In this task, like in standard change-of-location tasks, participants need to track where an agent believes an object to be that was re-located in her absence. But rather than using discrete locations (the object was in box 1 and then moved to box 2), the object is placed and re-located within a continuous space such as a sandbox. Participants can thus track more or less precisely where the object was and now is and where the agent believes it to be. This task has been used with participants of a wide age range (e.g., three- and five-year-olds; young, middle-aged, and senior adults) in the forms of real-object, paper-pencil, or computerized versions (Bernstein et al., 2011; Begeer et al., 2012; Sommerville et al., 2013; Coburn et al., 2015; Mahy et al., 2017; Samuel et al., 2018a,b). In the existing, explicit egocentric bias version, participants witness an object being re-located from Location 1 to Location 2 in the absence of the agent. Then they are asked where the agent would look for the object. Egocentric interference effects suggest that participants’ answers would be biased away from Location 1 (i.e., correct answer) in the direction of Location 2 (i.e., object’s current location) as they know that the object is now at Location 2. This task lends itself nicely to developing an analogous implicit or altercentric bias version. This new implicit version is just like the explicit version of the task, except for one crucial difference in the test question. In the altercentric bias version, participants are questioned on the object’s current location rather than where the agent would look for it. If they are subject to altercentric interference effects, their answers will deviate from Location 2 (i.e., correct answer) in the direction of Location 1 (i.e., agent’s belief location). The construction of closely matched explicit and implicit versions of the Sandbox FB task thus allows us to investigate and contrast egocentric and altercentric biases within the same task format in fine-grained ways. Here, we report three studies that explore the viability of such combined task formats with adults. All the studies reported in this paper were conducted online during and due to the Covid-19 pandemic.^1^

## 2. Study 1

The aim of Study 1 was to investigate whether an online adaptation of the Sandbox task could tap both egocentric and altercentric biases. This study also explored the presence and the magnitude of these biases in two different cultures (German/Western and Turkish/Eastern). Western societies are regarded as independent cultures as they emphasize attention to self and individualist self-construals. In contrary, Eastern cultures emphasize being attentive to others and harmony between individuals, leading to more interdependent self-construals (Markus and Kitayama, 1991). As a result of this difference, Western people could be subject to egocentric bias more than Eastern people as they operate with a focus on themselves. In contrast, Eastern people could be more prone to altercentric bias as they prioritize others’ perspectives above their own. Mixed results have been provided for this potential difference so far. For example, Wu and Keysar (2007) found that Chinese adults showed less egocentric bias than their American counterparts. By contrast, Wang et al. (2019) did not find any difference between Taiwanese and British adults in terms of egocentric and altercentric biases. These studies have either measured only one bias or measured the biases in separate tasks. The current study aims to explore potential differences in both egocentric and altercentric biases within one task format and with samples that have not been compared in this context before (i.e., German and Turkish samples). The study was preregistered.^2^

### 2.1. Method

#### 2.1.1. Participants

Participants were recruited through social media advertisements and e-mail announcements. All participants were tested online in unmoderated sessions via Qualtrics.^3^ We used G*POWER (Faul et al., 2009) to conduct a power analysis and determine the sample size. We aimed to obtain 0.95 power to detect a medium effect size of 0.54 at the standard 0.05 alpha error probability with a more conservative two-tailed paired-samples t-test. The effect size was based on an earlier study by Samuel et al. (2018b). They found a significant difference between experimental and control trials of the egocentric bias condition using a computerized version of the Sandbox task. Since the main aim of the current study was to tap biases revealed as the differences between experimental and control trials, we based the sample size rationale on within-subject comparisons rather than between-subject comparisons. The analysis revealed a required sample size of 47 participants for each bias measured within a group: 94 participants per group and 188 participants in total. Thus, the final sample consisted of 188 participants: 94 German (72 females, M_age_ = 28.11, age range: 18 to 62) and 94 Turkish (63 females, M_age_ = 27.05, age range: 18 to 57) adults. All participants were tested in their native languages, consented to the study, and, upon completing the study, became eligible for a lottery that distributed vouchers from online bookstores.

In order to have a final sample of 188 participants, we initially tested 356 participants. One hundred fifteen participants were excluded from the final sample as they did not complete all trials (M_CompletedTrials_ = 3.99, SD = 2.06, range: 1 to 7), 34 of them exceeded the time limit allocated for the study (i.e., 30 min), 16 of them had technical problems, and 3 of them reported that their native languages were different from the desired languages (i.e., German and Turkish).

#### 2.1.2. Materials

##### 2.1.2.1. The sandbox task

The scenarios used in our study were based on those used in Mahy et al. (2017). They always followed the same storyline: Agent A hides an object in Location 1, but then the object is transferred to Location 2 by Agent B either in the absence (False Belief) or in the presence of Agent A (True Belief). After the scenarios were presented, participants worked on a word-search puzzle for 20 s. Puzzles prevented using perceptual cues to answer the question and were created by inserting six randomly generated city names (from participants’ respective countries) into a 10 × 10 word-search puzzle using a puzzle maker website.^4^

The images presented to the participants (1,500 × 1,125 pixels) displayed a rectangular container (1,220 × 150 pixels) positioned in the middle of the image and text above the container. The crosses (37.5 × 37.5 pixels) on the container indicated a hidden object’s initial and final locations. These locations were always 746 pixels apart, but their relative position changed across trials to prevent participants from learning the locations. In all of our studies, the direction of relocation was counterbalanced: in half of the trials, the object was transferred from left to right, and in the other half, the transfer was from right to left. The objects always crossed the midline of the sandbox during the transposition.

**Figure 1:**
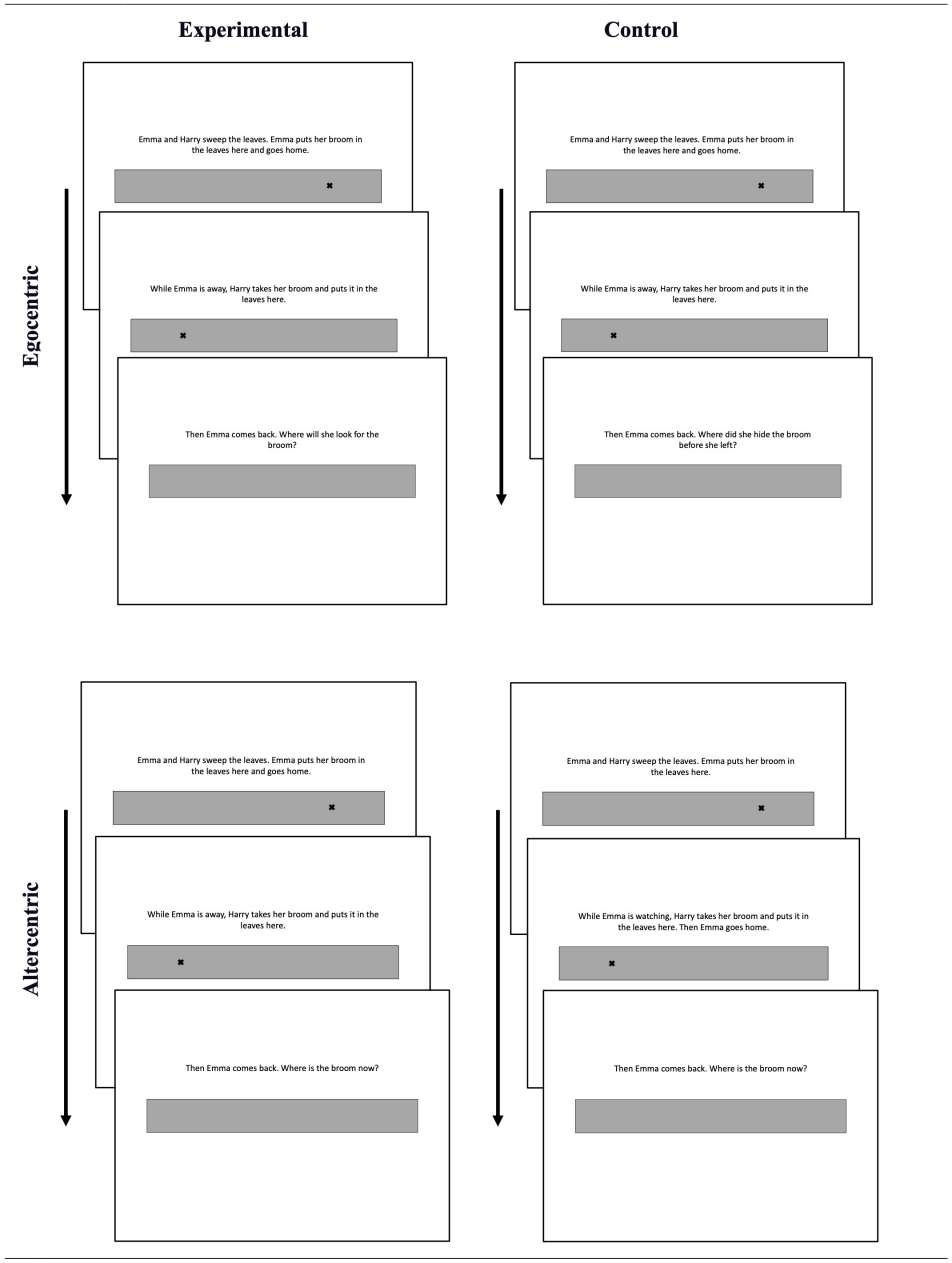
Sample materials from Study 1.

In a mixed design study, participants were presented with either the egocentric or altercentric bias conditions, consisting of experimental and control trials. These two conditions utilized the same task format and scenarios but differed in their test questions (see Figure 1 for examples). In the egocentric bias condition, participants were asked either where Agent A, who has a false belief about the object’s location, would look for the object upon return (experimental trials; “Where will X look for the object?”) or where s/he hid the object before leaving the scene (control trials; “Where did X hide the object?”). In both of these trials, the correct answer is around Location 1. Participants are expected to deviate in the direction of Location 2 in the experimental trials as they know that the object is actually at Location 2, and this knowledge is expected to interfere with their judgments of others’ perspectives and behaviors. In the altercentric bias condition, the test question always inquired where the object currently is, but the preceding scenario differed in terms of Agent A’s belief (which was irrelevant to the test question) in experimental and control trials. In the control trials, Agent A witnessed the relocation, therefore, had a true belief about the object’s current location. In the experimental trials, the relocation happened in Agent A’s absence; therefore, Agent A had a false belief about the object’s location. In both of these trials, the correct answer is around Location 2. Participants are expected to deviate in the direction of Location 1 in the experimental trials but not in the control trials, as the agent in the experimental trials thinks that the object is still at Location 1.

##### 2.1.2.2. Self-construal scale

In this 30-item Likert-type scale, participants evaluated the strength of their self-construal in terms of independency and interdependency (15 questions per each type of self-construal, Singelis, 1994; see Supplementary materials for example items). For our study, the original scale was translated and backtranslated by German and Turkish native speakers, who were also fluent in English. Each participant received two independence and interdependence scores ranging between 15 and 105.

##### 2.1.2.3. Demographic questionnaire

This questionnaire consisted of four questions asking the participants’ age, gender, highest educational degree achieved, and native language.

#### 2.1.3. Design and procedure

In a mixed design, Turkish and German participants were randomly assigned either to the egocentric or the altercentric bias condition, which included two types of trials: experimental and control. Each participant completed four experimental and four control trials presented in blocks (the order of the blocks counterbalanced) and one filler trial in between.

After consenting to the study, participants started test sessions with a calibration task. Participants were asked to click on the center of six crosses presented on the screen in this task. These crosses represented the endpoints of the Sandbox. Calibration trials provided information about mouse cursors’ sensitivity. Then the Sandbox task, the Self-Construal Scale, and the demographic questionnaire were completed in this fixed order. In the end, participants were debriefed about the real aim of the study and registered their contact information if they wanted to participate in the lottery. The study took approximately 20 min.

#### 2.1.4. Bias calculation and analysis

**Figure 2:**
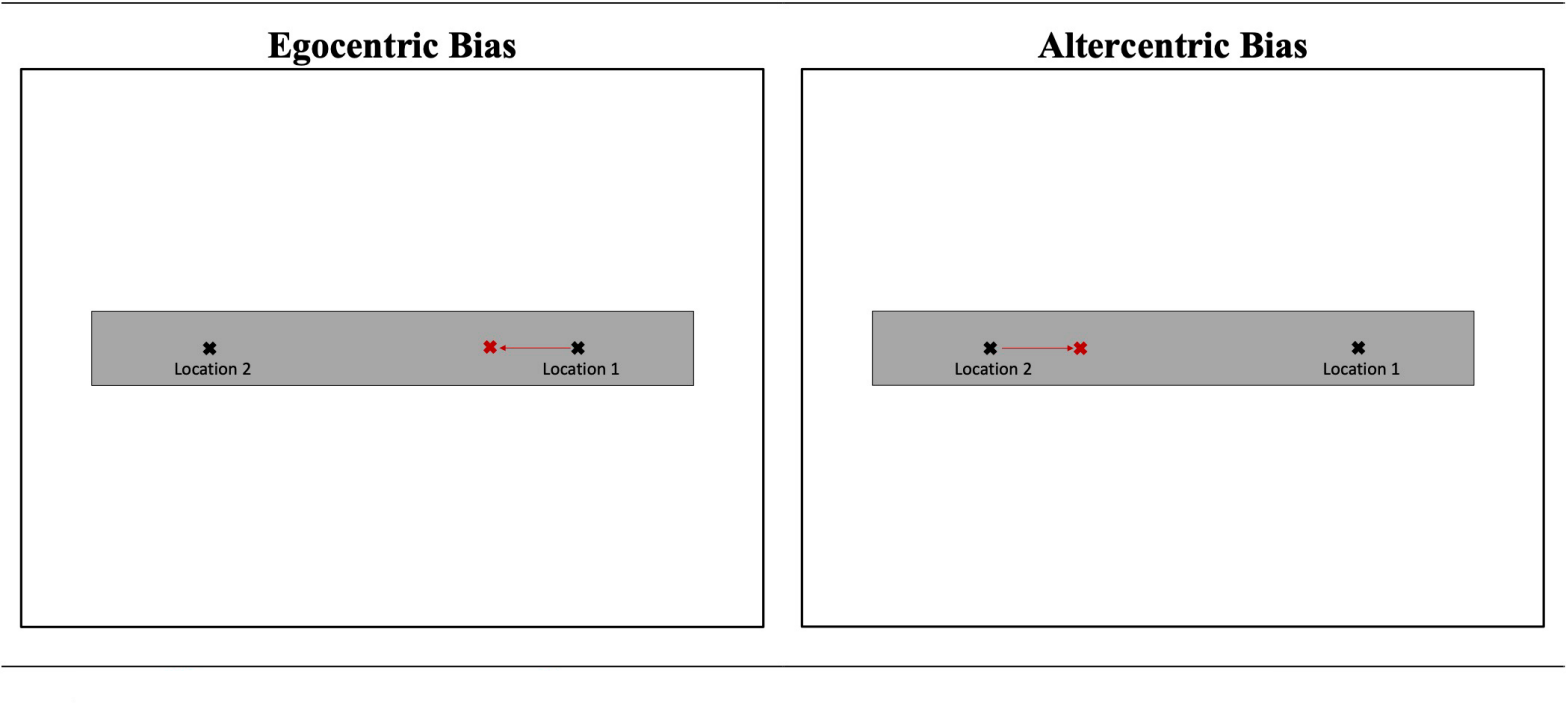
Bias calculation in Study 1. 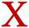: Participant’s answer. 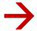: Object location measure.

Biases were inferred from the object location measure: the horizontal distance (in pixels) between the correct location (i.e., L1 in egocentric condition, L2 in altercentric condition) and the participant’s response (see Figure 2 for an illustration). If the participants’ responses were biased toward the wrong location (i.e., between the right and wrong answer, toward to middle of the screen), they received a positive object location value. The responses biased away from the wrong location (i.e., in the direction of the edge of the Sandbox/screen, rather than the middle) received a negative object location value. Once the object location measure was computed for each trial, we calculated the average object location measure in experimental and control trials for each participant. The averages were calculated in two ways: (a) all responses were included in the averages (as done in the original Sandbox task studies), and (b) the completely wrong answers (i.e., responses that were closer to the incorrect location than the correct location) were excluded from the averages. The latter method aimed to exclude the trials to which participants did not pay enough attention. We argue that adults are expected to have full-fledged perspective-taking abilities; therefore, completely wrong answers would reflect participants’ failures of attention and could be excluded from the data for explorative purposes (e.g., does a bias exist when only the attended trials are considered?). The average scores were then used to deduce biases: if the average deviation in the experimental trials is bigger than the control trials, this indicates bias. As a result, separate within-subject comparisons were conducted with and without wrong answers to see if a bias exists in different conditions and groups.

In addition to investigating whether a bias exist, we also explored whether German and Turkish participants differed in terms of (a) independency/interdependency and (b) the magnitude of any bias. To compare the biases shown by different groups, we created a pure bias score for each bias type by subtracting the average deviation in control trials from the average deviation in experimental trials. This score enabled us to compare the two groups directly on the difference between experimental and control trials, namely, the deviation expected due to the perspective-taking. Finally, we explored if pure bias scores were related to the level of independency and interdependency.

Following Samuel et al. (2018b), we used non-parametric tests (e.g., matched-pair Wilcoxon signed-rank Test, Mann–Whitney U test) for both within- and between-subject comparisons, as the response data were not normally distributed. For all of our studies, we also ran analyses with parametric tests as we had initially expected a continuous distribution of answers. The pattern of results and significances remained the same across all studies.

### 2.2. Results

#### 2.2.1. Within-subject comparisons: do the biases exist?

**Figure 3:**
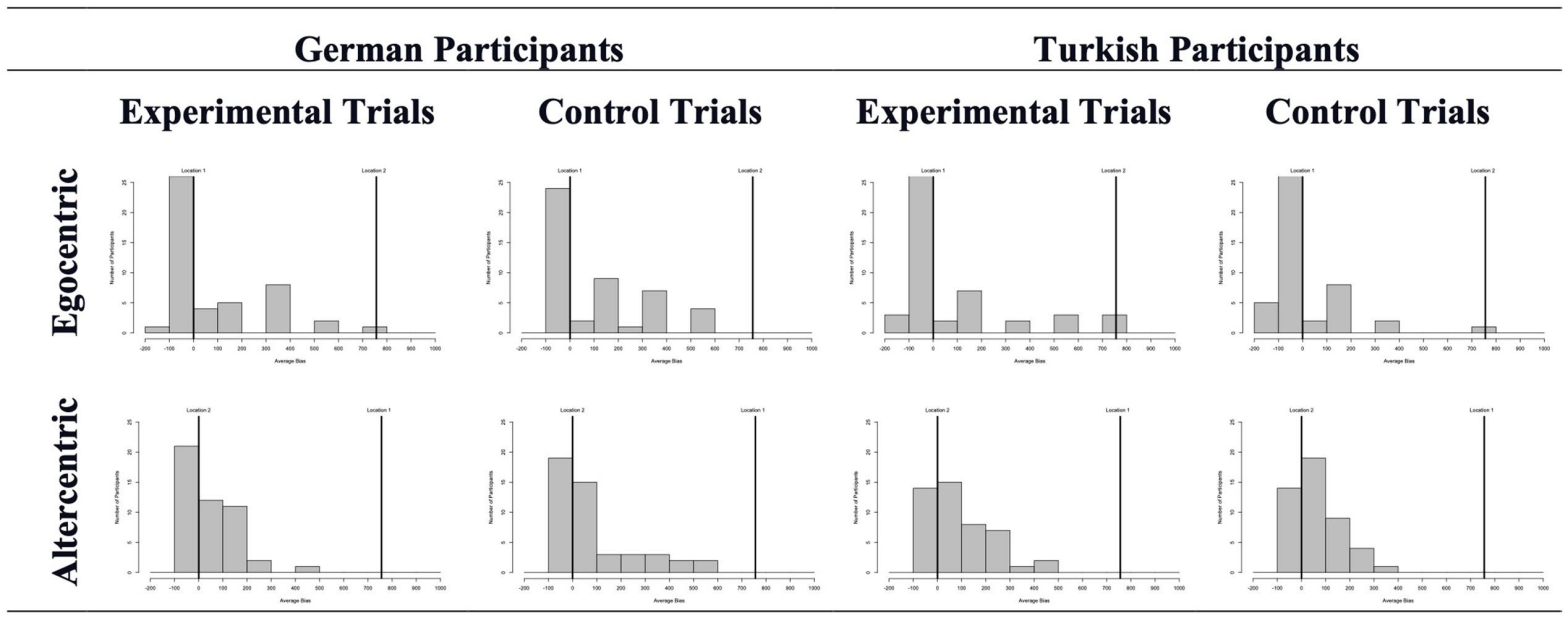
Average object location deviations in experimental and control trials across conditions and participants in Study 1. Here (and in the following studies) the binned data is presented for ease of depiction, but the analyses were run with the continous data.

The mean deviations in experimental and control trials can be seen in Figure 3. We started our analysis by comparing these average deviations across biases and groups. Almost none of these analyses revealed a difference between experimental and control trials. Specifically, the experimental vs. control trials differed in neither the egocentric (experimental: *Mdn* = −15.0; control: *Mdn* = −13.0) nor the altercentric bias (experimental: *Mdn* = 5.0; control: *Mdn* = 8.5) conditions for German adults, *Z* = -0.540, *p* = 0.589 and *Z* = -1.360, *p* = 0.174, respectively. Excluding the wrong answers from the analysis did not change the sample size as none of the participants failed all trials. The general picture drawn by the results remained the same too: experimental and control trials were very similar for both egocentric (experimental: *Mdn* = −39.63; control: *Mdn* = −47.0) and altercentric (experimental: *Mdn* = −15.0; control: *Mdn* = −3.25) bias conditions, *Z* = -1.830, *p* = 0.067 and *Z* = -1.423, *p* = 0.155, respectively. For Turkish participants, experimental (*Mdn* = −27.75) and control (*Mdn* = −39.5) trials only differed for the egocentric bias when all responses were included in the analysis, *Z* = -2.349, *p* = 0.019; but not after the wrong answers were excluded (experimental: *Mdn* = −45.0; control: *Mdn* = −51.375), *Z* = -1.654, *p* = 0.098. No altercentric bias was found regardless of calculation, i.e., with (experimental: *Mdn* = 28.75; control: *Mdn* = 6.5) or without (experimental: *Mdn* = −0.25; control: *Mdn* = 0.25) wrong answers, *Z* = -1.365, *p* = 0.172 and *Z* = -0.561, *p* = 0.575, respectively.

#### 2.2.2. Between-subject comparisons: do the groups differ?

We first compared German and Turkish adults in terms of the independency-interdependency scores to see if the two groups really differed. Results revealed a difference only in one domain: Turkish adults scored higher on the interdependency measure than German adults, *U* = 3105.50, *Z* = −3.520, *p* < 0.001; however, they did not differ in the independency sub-scale, *U* = 3756.50, *Z* = −1.775, *p* = 0.076. Then we explored if the two cultures differed in the pure bias shown in egocentric and altercentric conditions. Mann–Whitney tests revealed a difference neither for the pure egocentric nor the pure altercentric bias of German and Turkish adults, *U* = 990.00, *Z* = −0.178, *p* = 0.859, and *U* = 920.00, *Z* = −1.395, *p* = 0.163, respectively. Finally, we investigated whether any pure bias and a type of self-construal were related. Results revealed no significant correlations (all *r*s = < |10|, all *p*s > = 0.09).

#### 2.2.3. Within-subject comparisons with collapsed datasets

As suggested by one of the anonymous reviewers, in order to increase power, we repeated the within-subject comparisons by collapsing the two groups since they did not differ. The analysis revealed a difference between experimental (*Mdn* = −41.0) and control (*Mdn* = −47.67) trials for egocentric bias (*Z* = -2.559, *p* = 0.01) only when the wrong answers were excluded from the analysis; but not for the whole sample (experimental: *Mdn* = −22.38, control: *Mdn* = −27.5; *Z* = -1.245, *p* = 0.21). No difference was observed in altercentric bias condition, regardless of the fact that the wrong answers were included (experimental: *Mdn* = −5.08, control: *Mdn* = −2.88; *Z* = -0.064, *p* = 0.95) or excluded (experimental: *Mdn* = 19.63, control: *Mdn* = 7.88; *Z* = -0.601, *p* = 0.55).

### 2.3. Discussion

Study 1 revealed almost no differences between experimental and control trials (except the collapsed analyses, where deviations were negative and were not biased toward the second location); hence no evidence for egocentric or altercentric biases and did not reveal any cross-cultural differences in egocentric and altercentric biases either.

The null results found in Study 1 are difficult to interpret, and they should be evaluated with caution for two reasons. First, there was a very high dropout rate (almost 50%). Even though some of these dropouts occurred due to technical issues or timeout, many participants intentionally stopped participating without completing the study simply because they were bored due to the dull materials. We suspect that the not-so-engaging task materials might have caused our remaining participants to fail to pay enough attention to the task, which could have made the task less reliable. Secondly, the altercentric bias measure may have not been spontaneous enough to tap automatic interference effects. Possibly, with too much time, participants begin to reflect on and evaluate their own perspective and correct any potential spontaneous biases. Therefore, although, in theory, we expected the Sandbox task to tap altercentric biases as well as egocentric biases, this task may not be suitable to detect altercentric interferences. These two issues were addressed in the following studies.

## 3. Study 2

In response to the potential shortcomings of Study 1, Study 2 used more engaging materials in the form of animated videos and presented fewer trials (i.e., two trials per trial type instead of four). Also, to have more spontaneous behavioral measures, the present study combined the Sandbox task with mouse-tracking measures. These measures have previously been used to document altercentric bias (e.g., Van der Wel et al., 2014). When subjects were asked to move their mouse cursors to the target object’s location, they took a little detour on their way to their answers when another agent in the scenario had a belief that differs from their own. However, when the participant and the agent shared a belief, participants followed a more direct route while moving their mouse cursors to mark the target location. Therefore, the area between the detour in the direction of the wrong answer and the direct line from the starting point to the target location indicated whether and to what degree participants engaged in altercentric bias. Since mouse-tracking measure is inferred from spontaneous motor responses, it constitutes a more suitable alternative to tap implicit biases, especially altercentric bias, than the object location measure of the Sandbox task. More specifically, this task is less subject to reflections and evaluations as it is not about the content of the final judgment, and it does not respond to an explicit trigger. Rather it occurs spontaneously and is manifested via automatic motor responses. Therefore, these measures are optimally suited to reveal online processes which are more automatic and spontaneous, such as altercentric interference effects, and could tap these biases more reliably (Van der Wel et al., 2014). The study was preregistered.^5^

### 3.1. Method

#### 3.1.1. Participants

In Study 2, we tested both English- and German-speaking adults online. These two groups were included not because we expected to see a difference but because of practical reasons. Namely, the platform where the study was published had a bigger pool of English-speaking participants, which meant more representative data. English- and German-speaking participants were presented with the same materials, except the language of the materials and instructions. The experimenter translated the materials, which were then double-checked by a native English speaker, who also did the voice recordings for English materials. Participants were recruited through Prolific^6^ and they were tested in unmoderated sessions via Labvanced.^7^ The sample size reasoning was the same as in the first study. Therefore, we collected 47 participants for each bias measured within a group, 94 participants per group and 188 participants in total. More specifically, we tested 94 German-speaking (34 females, M_age_ = 30.60, age range: 18 to 63) and 94 English-speaking (52 females, M_age_ = 33.14, age range: 18 to 65) adults. All participants were tested in their native languages, consented to the study, and received compensation upon completing it. At the beginning of the study, participants were questioned on their demographic information, including age, gender, education level, and native language.

In order to have a final sample of 188 participants, we initially tested 198 participants. Five participants were excluded from the final sample as they had technical issues, 3 of them did not complete all trials (M_CompletedTrials_ = 2.67, SD = 0.58, range: 2 to 3), and 2 of them exceeded the time limit allocated for the study (i.e., 20 min).

#### 3.1.2. Materials

**Figure 4:**
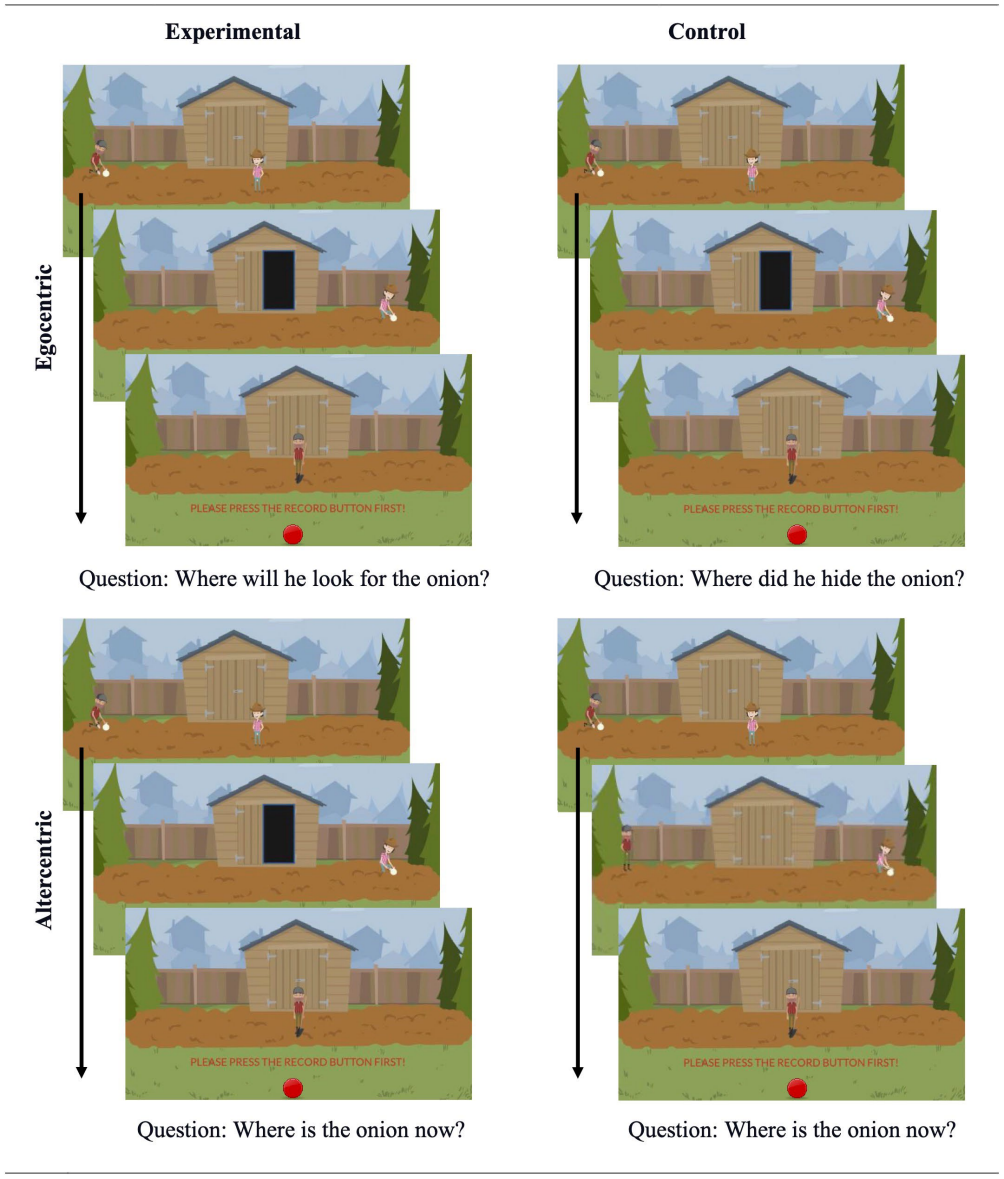
Sample materials from Studies 2 and 3.

The scenarios used in the second study were similar to the first study. They always featured two agents and an object, and they followed the same storyline: Agent A hides an object in Location 1, but then the object is transferred to Location 2 by Agent B either in the absence (False Belief) or the presence of Agent A (True Belief). After the videos were presented, participants searched and marked dots on the screen for 10 s. This task aimed to prevent participants from using perceptual cues to answer the question and served as a calibration check. This task was created directly on Labvanced. It generated colorful dots (20 × 20 units) and presented them on the screen one by one. Participants were instructed to find and click on these dots. Besides the already mentioned differences, the scenarios and questions used in the control and experimental trials of Study 2 closely resembled Study 1. Figure 4 depicts the important sections of the videos used in Study 2 (and Study 3).

The materials used in the second study were different from the first study in several ways. First, in Study 2, the stories were presented as animated videos instead of still pictures. Second, participants did not see any sandbox drawing with borders. Instead, the whole screen was used as the hiding area, and there was no visual cue that could potentially cause anchoring effects. Third, we opted for a simpler distractor. More specifically, participants were asked to click on dots that appeared on the screen instead of word-search puzzles. Also, in Study 2, the distraction task lasted 10 s instead of 20 s. This distractor task also allowed us to check the calibration of their mouse cursors throughout the experiment and control it if necessary. Fourth, we decreased the number of trials per condition. Participants answered two experimental and two control trials, with one filler trial in between. Finally, adding the mouse-tracking measures called for a small but crucial change in the materials. As in Study 1, participants were asked the test question after completing the distraction task. Unlike Study 1, however, they first needed to press a record button located at the bottom middle of the screen to be eligible for responding to test questions. Only then were they able to do any marking on the screen. This procedure ensured that all participants started to move their mouse cursors at the same point on the screen.

The videos presented to the participants were created in Vyond,^8^ then converted into the movie format, and uploaded to Labvanced. The videos (800 × 450 units) were always presented in landscape format. The hiding locations were always 557.5 units apart, which is almost 70% of the screen. Their relative position changed on the same horizontal line across trials to prevent participants from learning the locations.

#### 3.1.3. Design and procedure

In a mixed design, English- and German-speaking participants were randomly assigned either to the egocentric or the altercentric bias condition, which included two types of trials: experimental and control. Each participant completed two experimental and two control trials presented in blocks (the order of the blocks counterbalanced) and one filler trial in between.

After consenting to the study, participants started test sessions with practice trials, where participants were asked to click on the center of two crosses presented on the screen. These crosses appeared at random locations, but their places were consistent across participants. These practice trials provided information about the sensitivity of mouse cursors and taught participants that they should start moving their cursors from a designated point. Namely, to be able to click on a cross, participants were required to first click on a record button, which was always presented at the bottom middle of the screen. This record button corresponded to the starting point of the mouse-tracking measure in later trials. After completing the practice trials, participants indicated which device was used to mark locations: a built-in touchpad or a mouse. Then they started the actual trials of the Sandbox task. In the end, participants were directed back to Prolific and received their payments. The study took approximately 12 min.

#### 3.1.4. Bias calculation and analysis

**Figure 5:**
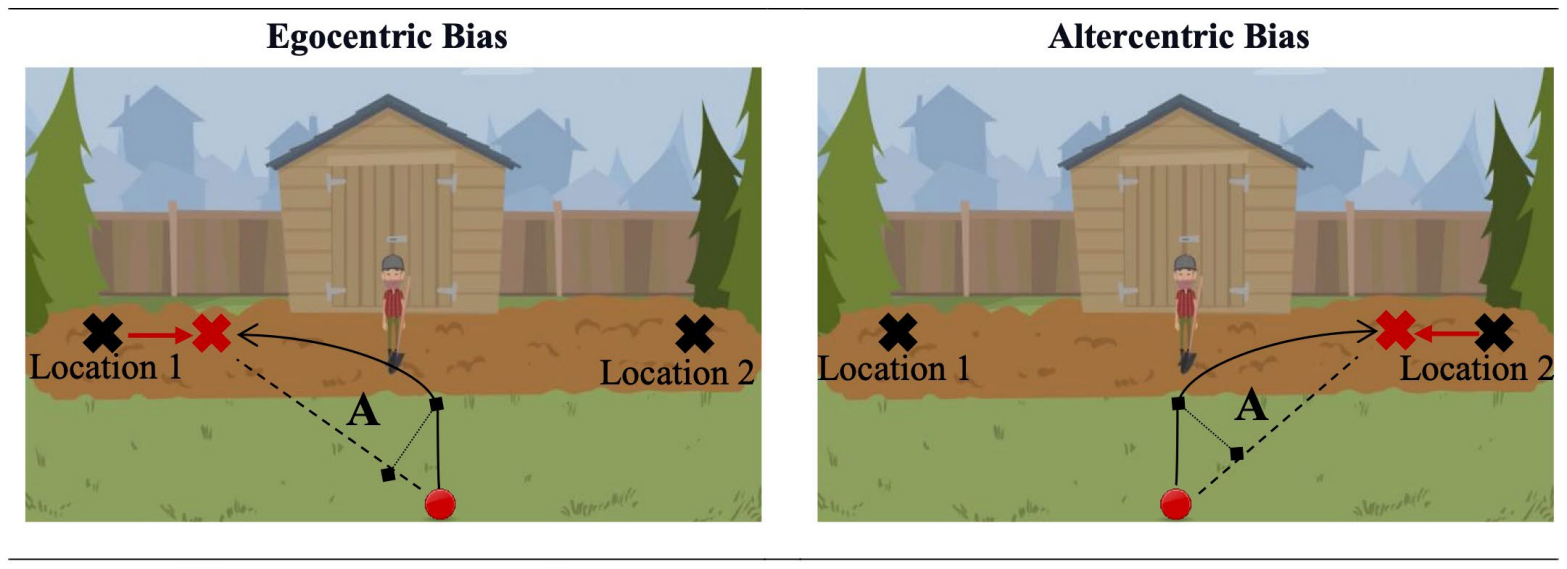
Bias calculation in Studies 2 and 3. 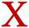: Participant’s answer. 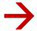: Object location measure, −−−: direct path to the participant’s answer. 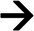: Participant’s mouse trajectory. A: mouse tracking measure. ♦−−−♦: an example orthogonal line drawn from the mouse coordinates to the direct path.

Bias scores were calculated in two ways in this study (see Figure 5). The first way was the object location measure as calculated in Study 1 (i.e., the horizontal distance between the correct location and the participant’s response), which was then used to calculate the average biases in experimental and control trials with and without wrong answers. The other way to measure egocentric and altercentric biases was to track the mouse movements of the participants. For this measure, we calculated how many units of area were between the path the participants followed on the way to their answers and the most direct (shortest) path to their answers. More specifically, Labvanced provided us with the time series and the coordinates of participants’ mouse movements on a standardized 800×450 unit surface area. These coordinates were then used to infer the actual trajectories followed by the participants, and the start and end points of the trajectories were used to compute the direct path to their answers. Because the trajectories varied in duration, they were standardized into 100 time steps and 100 coordinates using linear interpolation. Then, orthogonal lines were drawn from each coordinate [(e.g., x_1_, y_1_) to the direct path (e.g., x_2_, y_2_)] and their lengths were calculated by using the distance formula [d = √ ((x_2_ – x_1_) ^2^ + (y_2_ – y_1_) ^2^)]. Then the length of every possible line was summed up to get the area between the actual trajectory and direct path for each trial. If participants showed a detour toward the wrong answer (i.e., Location 2 in egocentric bias and Location 1 in altercentric bias), the area between this detour and the direct route is assigned a positive value as this detour indicated that participants were biased. If participants show a detour in the opposite direction (i.e., between the direct path and the edge of the screen) the area is given a negative value. Then the average mouse trajectory measure was calculated for experimental and control trials in each condition. The mouse-tracking measure was only calculated for correct answers; therefore, only one average value was obtained per trial type (experimental versus control).

Finally, similar to the first study, separate within-subject comparisons were conducted for egocentric and altercentric bias conditions and English- and German-speaking participants to see if experimental and control trials differ; hence a bias exists. Following Samuel et al. (2018b) and the first study of this paper, we used non-parametric tests (i.e., matched-pair Wilcoxon signed-rank Test) to conduct within-subject comparisons as the response data were not normally distributed.

### 3.2. Results

**Figure 6:**
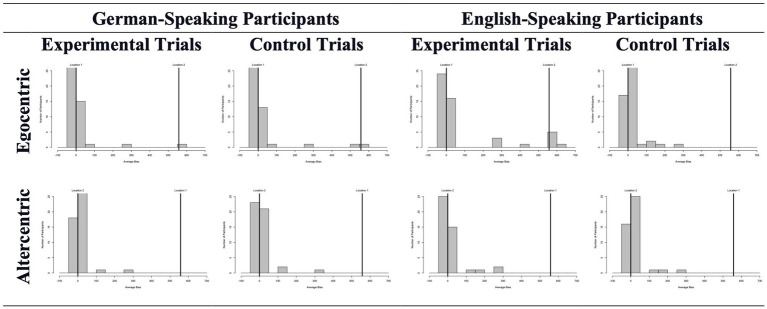
Average object location deviations in experimental and control trials across conditions and participants in Study 2.

The mean object location deviations in experimental and control trials are depicted in Figure 6. We first separately compared the average biases in experimental and control trials for each condition (i.e., egocentric and altercentric) and group (German- and English-speaking adults). None of these analyses suggested a difference between experimental and control trials; hence no bias was revealed. As object-location measures have shown, the experimental versus control trials differed in neither the egocentric (experimental: *Mdn* = −4.17; control: *Mdn* = −5.51) nor the altercentric (experimental: *Mdn* = 1.88; control: *Mdn* = 1.56) bias conditions for German-speaking adults, *Z* = −1.376, *p* = 0.169 and *Z* = −0.317, *p* = 0.751, respectively. Excluding the wrong answers from the analysis did not change the direction of these results: experimental and control trials were not different from each other, neither for egocentric (experimental: *Mdn* = −5.23; control: *Mdn* = −6.2) nor altercentric (experimental: *Mdn* = 1.51; control: *Mdn* = −0.94) bias conditions, *Z = −0.993, p = 0.321* and *Z = −0.709, p = 0.478*, respectively. The mouse-tracking measure revealed similar results: mouse movements in experimental and control trials resembled each other both in egocentric (experimental: *Mdn* = 3.23; control: *Mdn* = 3.87) and altercentric (experimental: *Mdn* = 3.75; control: *Mdn* = 3.73) bias conditions: *Z* = −0.672, *p* = 0.502 and *Z* = −0.550, *p* = 0.582, respectively.

The results obtained from English-speaking participants were in line with those from German-speaking adults. Accordingly, the object-location measure did not reveal any difference between experimental and control trials, neither in the egocentric (experimental: *Mdn* = 1.79; control: *Mdn* = 5.52) nor the altercentric (experimental: *Mdn* = −0.71; control: *Mdn* = 3.09) bias conditions: *Z* = -0.492, *p* = 0.622 and *Z* = -1.144, *p* = 0.253. The results remained almost the same after excluding the wrong answers: the difference between trial types approached significance in the egocentric bias version (experimental: *Mdn* = −4.38; control: *Mdn* = 4.53), *Z* = -1.781, *p* = 0.075; however, no such pattern has been shown for the altercentric bias condition (experimental: *Mdn* = −1.24; control: *Mdn* = 3.09), *Z* = -1.453, *p* = 0.146. Mouse-tracking measures did not reveal any difference, neither for egocentric (experimental: *Mdn* = 2.88; control: *Mdn* = 4.78) nor altercentric (experimental: *Mdn* = −1.24; control: *Mdn* = 3.09) bias conditions, *Z* = -1.319, *p* = 0.187 and *Z* = -0.455, *p* = 0.649, respectively.

Similar to Study 1, in order to increase power, we repeated the within-subject comparisons by collapsing the two language groups. First, we checked if the groups differed in terms of the pure biases they showed. No differences were observed between groups. Then we repeated the within-subject comparisons with the entire dataset of Study 2 and found no difference between experimental and control trials regardless of the measure, bias condition, and whether the wrong answers were excluded or not. More specifically, object-location measures have shown that the experimental and control trials differed in neither the egocentric (experimental: *Mdn* = −4.17; control: *Mdn* = −1.62) nor the altercentric (experimental: *Mdn* = 1.25; control: *Mdn* = 2.81) bias conditions, *Z* = −0.480, *p* = 0.63 and *Z* = −0.564, *p* = 0.57, respectively. Excluding the wrong answers from the analysis did not change these results: experimental and control trials were not different from each other, neither for egocentric (experimental: *Mdn* = −4.58; control: *Mdn* = −2.24) nor altercentric (experimental: *Mdn* = 0.42; control: *Mdn* = 2.29) bias conditions, *Z = −1.847, p = 0.07* and *Z = −0.544, p = 0.59*, respectively. The mouse-tracking measure revealed similar results: mouse movements in experimental and control trials resembled each other both in egocentric (experimental: *Mdn* = 2.88; control: *Mdn* = 3.98) and altercentric (experimental: *Mdn* = 3.86; control: *Mdn* = 3.23) bias conditions: *Z* = −1.348, *p* = 0.18 and *Z* = −0.716, *p* = 0.47, respectively.

### 3.3. Discussion

Study 2 was successful in the sense that the dropout rate declined considerably (5%) compared to Study 1. However, there was still no evidence for any bias in the previously used (i.e., object location) or in the newly added (i.e., mouse tracking) measure. There is still one possibility that remains open and might provide some potential explanations of the null results: socio-cognitive biases may be sensitive to test designs and procedures and reveal themselves only under specific circumstances. One such design could be within-subject studies where participants are tested on both biases in blocks. Existing evidence is compatible with the possibility that altercentric biases only arise in such mixed-block designs (Furlanetto et al., 2016; Speiger et al., 2022) and not in analogous single-block designs like the one used in Study 2 (e.g., Conway et al., 2017). We investigated this possibility in Study 3.

## 4. Study 3

Study 3 used the Sandbox task as in Study 2, including both object location and mouse-tracking measures in a within-subjects design with mixed altercentric and egocentric bias blocks. The study was preregistered.^9^

### 4.1. Method

#### 4.1.1. Participants

Fifty-four German-speaking adults (18 females, M_age_ = 28.92, age range: 18 to 66) were tested via Prolific on an online study created through Labvanced. Since the demographics data provided in Study 2 showed that Prolific had access to a representative German-speaking sample, Study 3 tested only German-speaking adults. We used G*POWER (Faul et al., 2009) to conduct a power analysis and determine the sample size. Our goal was to obtain 0.95 power to detect a medium effect size of 0.50 at the standard 0.05 alpha error probability in a MANOVA, in which between- and within-subject comparisons would be conducted with both object location and mouse-tracking measures. This sample size also matched the sample size of Conway et al. (2017) study, where an altercentric bias was found only with a within-subject design. Participants were tested in German, consented to the study, and received compensation upon completing the study. At the beginning of the study, participants were questioned on their demographic information, including age, gender, education level, and native language.

Only one participant was excluded from the initial data set due to incomplete trials (only three trials were completed by this participant).

#### 4.1.2. Materials

The materials used in Study 3 were the same as in Study 2, except that Study 3 included more trials because of the within-subject design and, therefore, more stories than Study 2. Based on the same storyline, four other videos were created for Study 3, again in Vyond. All stories featured two agents, one object, and relocation of the object either in the agent’s absence or presence. We did not change anything regarding the practice trials, the distractor task, and the requirement of pressing the record button before answering. We also kept the format of the experimental and control trials the same for both egocentric and altercentric bias conditions.

#### 4.1.3. Design and procedure

In a mixed design, participants were tested on both egocentric and altercentric biases. They were randomly assigned either to the egocentric-first or the altercentric-first condition. Both egocentric and altercentric bias measures included two types of critical trials: experimental and control. Each participant completed two experimental and two control trials presented in blocks per bias (the order of the blocks counterbalanced). This resulted in eight trials in total. Apart from the within-subject testing of the biases, the procedure of Study 3 was the same as in Study 2. The study took approximately 15 min.

#### 4.1.4. Bias calculation and analysis

The biases were calculated in the same way as in Study 2. Experimental and control trials were compared again with non-parametric Mann–Whitney U tests (due to the failure of the normality assumption). We also utilized non-parametric Wilcoxon-Signed Rank tests for between-subject comparisons. Although this analysis is not following the preregistered analysis on which the sample size was based, non-parametric tests were deemed more appropriate as the data were not normally distributed.

### 4.2. Results and discussion

**Figure 7:**
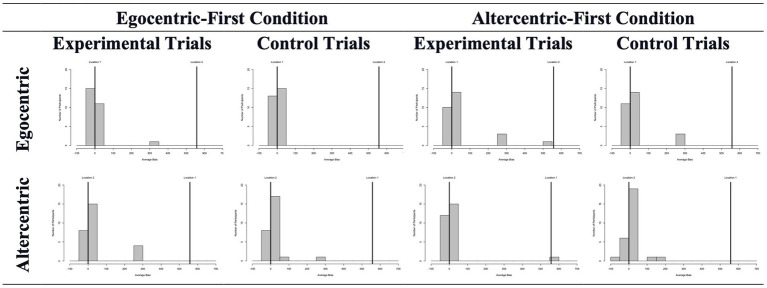
Average object location deviations in experimental and control trials across conditions and participants in Study 3.

The mean object location deviations in experimental and control trials are shown in Figure 7. We first compared these averages for each bias separately. For egocentric bias as measured by the Sandbox task, experimental (*Mdn* = 2.88) and control (*Mdn* = 3.27) trials did not differ from each other*, Z* = -0.030, *p* = 0.976; and excluding the wrong answers did not change this result, *Z* = -0.598, *p* = 0.550 (experimental trials: *Mdn* = 1.5; control trials: *Mdn =* 3.27). Mouse-tracking measures provided results along the same lines: no difference was found between experimental (*Mdn* = 1.47) and control (*Mdn* = 0.63) trials, Z = -1.536, *p* = 0.125. The altercentric bias version did not reveal any difference either. When all answers were considered, experimental (*Mdn* = 3.31) and control (*Mdn* = 7.9) trials did not differ from each other, Z = -0.697, *p* = 0.486. When the analysis was repeated with correct answers only, there was still no difference between experimental (*Mdn* = 2.71) and control (*Mdn* = 7.06) trials, Z = -1.54, *p* = 0.123. Mouse-tracking measures did not reveal any difference between trial types, *Z* = -1.102, *p* = 0.270 (experimental trials: *Mdn* = 2.56; control trials: *Mdn* = 3.19). We also compared the experimental and control trials separately in the altercentric-first and egocentric-first conditions. None of these comparisons revealed a difference (all *p*s > = 0.06).

When investigating possible carry-over costs between biases, no difference was found as a function of the presentation order. For pure egocentric bias measured by the Sandbox task, a Mann–Whitney test revealed no difference between egocentric-first vs. altercentric-first conditions, *U* = 320.00, *Z* = −0.552, *p* = 0.581. Mouse-tracking measures revealed similar null results, *U* = 278.00, *Z* = −1.299, *p* = 0.194. As to pure altercentric bias, neither the Sandbox nor the mouse-tracking measure revealed any difference between egocentric-first versus altercentric-first conditions; *U* = 278.00, *Z* = −1.299, *p* = 0.194 and *U* = 323.00, *Z* = −498, *p* = 0.618, respectively.

Overall, the present study thus failed to find any evidence for egocentric or altercentric biases even in a within-subjects block design. There was also no evidence for an effect of the order of blocks presented.

## 5. General discussion

The present study developed a new task to test egocentric and altercentric biases, as potential indicators of explicit and implicit ToM, within the same task format. To this end, building on existing continuous explicit False Belief (Sandbox) tasks, closely matched altercentric and egocentric versions of an online task were devised. Across three studies and two different measures, we found no evidence for any bias. More formal investigation of the null results via Bayes Factors analyses yielded mostly anecdotal to moderate evidence for the null hypotheses across all studies and conditions (with minor exceptions).^10^ Even though the experimental and control trials differed from each other in the collapsed analyses in Study 1, the deviations were not in the expected direction and thus do not reveal a true bias of interest. In addition, there was no evidence for cross-cultural differences (Study 1) or the effect of order of task versions administered (Study 3). In the following, we discuss whether this absence of evidence may constitute evidence of absence or merely false negatives.

### 5.1. Absence of evidence or evidence of absence for egocentric bias?

So far, original studies of the Sandbox task have repeatedly revealed significant egocentric interference effects for both children and adults (e.g., Bernstein et al., 2011; Begeer et al., 2012; Sommerville et al., 2013; Coburn et al., 2015; Mahy et al., 2017). These positive findings have been challenged by more recent replication attempts (Samuel et al., 2018a,b), where the egocentric interference effects were either absent or may have occurred due to a general difficulty with reasoning about false representations rather than false beliefs. As an example for the latter, Samuel et al. (2018a) have found equivalent levels of egocentric bias when participants were asked to indicate where a false film would depict an object as when they were asked about a protagonist’s false belief regarding the object’s location. The results of the current study add to the unsuccessful replication attempts and null results. It should be noted that the current study constitutes a conceptual, rather than a direct, replication attempt. Following Machery (2020), we do not argue that one form of replication is more valuable than the other. We simply emphasize that the current study was different than the original studies in terms of the task format and visual materials (starting from Study 2); and it aimed to extend the original studies to various samples by using additional measures.

But why do some studies succeed in finding evidence for egocentric biases whereas others do not? Are there any deep and systematic differences that can explain this pattern of positive versus null findings? One such potential difference may lie in the format of the studies: These differences between in-person versus online tasks could occur due to various reasons such as video-deficit effect, which has been shown to influence children’s performance on FB tasks (e.g., Reiß et al., 2019) or decreased attention and motivation during online testing (see for their possible hindering effects in memory tasks, Finley and Penningroth, 2015). The two pilot studies we have conducted speak against these possibilities and extend the null results to an in-person (paper-pencil) version of the Sandbox task (see Supplementary Documents). However, those pilot studies were not direct and systematic comparisons of the online versions we used, therefore they should be approached with caution. And these possibilities should be systematically tested in the future studies where the live versions are directly compared with the online version of the task.

First systematic comparisons of live vs. online studies have recently been conducted in socio-cognitive developmental research with children, with somewhat mixed findings. For example, Schidelko et al. (2021) found no difference between lab versus online versions of standard False Beliefs tasks, while Sheskin and Keil (2018) found considerable differences between the two versions (with much poorer performance in online FB tasks). This kind of systematic comparisons should be extended to adult samples and measures such as the Sandbox task before we can conclusively interpret null findings like the present one.

Another open question about the Sandbox task is whether it is subject to domain-general reasoning strategies that can explain the biases without any reference to perspectives. For example, it is possible that in the existing egocentric bias version of this task, participants are biased toward the second location merely because they are drawn to the presence of an alternative location, but not because they are influenced by their own perspective. This would then cause participants to be biased to the second location equally in the experimental and control trials; hence, no difference should then be detectable between these trials. These concerns are not relevant for the present work as participants either showed negative biases or binary response patterns in the current studies (i.e., they were not biased toward the second location). However, future studies using the Sandbox task should take preventive precautions for this kind of alternative explanations. For example, adding a nonmental control condition (e.g., objects are moved by the wind rather than agents) would reveal if participants are biased to the incorrect locations just because these locations exist (i.e., participants would be drawn to the second location in both mental and nonmental conditions) or because they are biased by their own perspectives (i.e., participants would be drawn to the second location only in the mental condition).

### 5.2. Absence of evidence or evidence of absence for altercentric bias?

When it comes to the altercentric interference effects, the null results in our studies are even more difficult to interpret. The adaptation of the implicit altercentric bias version of the Sandbox task used in the current study is completely new and exploratory; hence, there is no existing body of positive or null findings to which we can compare the present results.

In theory, different factors could be at play in terms of the existing null results. One set of factors that could make a difference is the superficial methodological factors such as the online format, boring and easy tasks, and technical limitations. More specifically, the unmoderated online format of the task and not-so-engaging materials might have caused inattention to task, leaving the differences between experimental and control trials undetected by the participants. It is also possible that participants found our altercentric bias version of the Sandbox task extremely easy, leading to ceiling effects in both experimental and control trials and making them indistinguishable from each other. Finally, technical limitations rendered the mouse tracking measures not-so-spontaneous in our studies. To ensure that all participants started moving their mouse cursors from the same point, we asked them to click on a “record button” before moving their mouse cursors. It is possible that this requirement interfered with more spontaneous and automatic altercentric bias effects and gave participants more time to reflect on their answers, leading to null results revealed by mouse movements.

There are also more substantial factors that could result in null altercentric interference effects. For example, it is possible that altercentric biases can be reliably found only in some domains for some types of measures but not in others. This bias is hypothesized to be automatic and spontaneous processing of others’ perspectives (e.g., Southgate, 2020). Therefore, more spontaneous temporal measures such as response times integrated into simpler tasks such as Level-1 visual perspective taking could reveal this bias better than the fine-grained spatial deviations in the contents of judgments about an object’s location. The latter would require more extended processing due to the preceding scenarios and its answer format whereas the former is more suitable for quick, automatic, spontaneous judgments. These factors should be explored in future research more systematically before the current task format is given up as a potential measure to tap altercentric bias.

### 5.3. Future directions and conclusion

Overall, the current set of studies thus failed to provide evidence for egocentric or altercentric biases in a novel combined task format. These null findings remain difficult to interpret and raise more questions for future research than they answer. In particular, it remains unclear whether the absence of evidence for egocentric and altercentric biases reflects the fact that these biases may be less robust than previously assumed. Alternatively, it could be that the biases are robust, but the present tasks (due to their online format or the specific content) are not suitable for tapping them. Systematic future investigations are required to answer these questions.

## Data availability statement

The raw data supporting the conclusions of this article will be made available by the authors, without undue reservation.

## Ethics statement

Ethical review and approval was not required for the study on human participants in accordance with the local legislation and institutional requirements. The patients/participants provided their written informed consent to participate in this study.

## Author contributions

FH, MP, UL, and HR contributed to conception and design of the study. FH conducted the studies, performed the statistical analysis, and wrote the first draft of the manuscript. MP, UL, and HR gave critical review and commentary on the drafts of the manuscript. MP and HR supervised the planning and execution process. HR provided resources for the data collection. All authors contributed to the article and approved the submitted version.

## Funding

This work was supported by Deutsche Forschungsgemeinschaft [Crossing the Borders Project, (LI 1989/3–2)] and by Research Training Group 2070 (Grant/Award Number: 254142454/GRK 2070). We also acknowledge support by the Open Access Publication Funds of the Göttingen University.

## Conflict of interest

The authors declare that the research was conducted in the absence of any commercial or financial relationships that could be construed as a potential conflict of interest.

## Publisher’s note

All claims expressed in this article are solely those of the authors and do not necessarily represent those of their affiliated organizations, or those of the publisher, the editors and the reviewers. Any product that may be evaluated in this article, or claim that may be made by its manufacturer, is not guaranteed or endorsed by the publisher.

## Footnotes

^1^We had started a pilot study that aimed to replicate earlier Sandbox task results (which revealed egocentric interference effects) using the paper-pencil version of the task and the original materials from this version. We had to stop the data collection for this version before reaching the necessary sample size due to the pandemic and we switched to online testing. The data collection for the pilot study has been recently completed. We also conducted a second pilot with more engaging materials. These are reported in the Supplementary Documents.

^2^ https://osf.io/36exv

^3^ https://www.qualtrics.com

^4^ https://puzzlemaker.discoveryeducation.com

^5^ https://osf.io/rqxs8

^6^ https://www.prolific.co

^7^ https://www.labvanced.com

^8^ https://www.vyond.com

^9^ https://osf.io/e5a23

^10^Following the suggestion of one anonymous reviewer, we also conducted separate one-sample Bayesian t-tests for each comparison we did in this study. In these Bayesian t-tests, we investigated if the data supported the null hypothesis (i.e., no difference exists between experimental and control trials). Following Dienes (2014), we accepted the BF_10_ value of 0.33 or smaller as a benchmark of a null result of sufficient sensitivity. BF_10_ values of 0.33 or below suggest that the data are at least three times as likely under the null hypothesis than under the alternative. In all one-sample Bayesian t-tests analyses, we used the difference between the experimental and control trials. This strategy was adopted from Samuel et al. (2018b). Almost all of these analyses revealed anecdotal to moderate evidence for null hypothesis, with Bayes factors ranging between 0.85 and 0.12 (indicating that the data were 1.12 to 8.68 times more likely under the null hypothesis). Only two analyses (Study 1 Turkish participants and Study 2 English-speaking participants, egocentric bias condition, when all answers were included in the compared data) provided anecdotal evidence for a difference between experimental and control trials, with a Bayes Factor of 2.53 in both cases.
