# Supplementary material for: How robust are egocentric and altercentric interference effects in social cognition? a test with explicit and implicit versions of a continuous false belief task
Source: Front Psychol. 2023 Jul 10;14:1142302. doi: 10.3389/fpsyg.2023.1142302 (PMC10363613; doi:10.3389/fpsyg.2023.1142302)
Supplement: Supplementary file 1 [file Data_Sheet_1.pdf]

Pilot Studies (Paper-Pencil Version of the Sandbox Task)

These studies aimed to investigate if the previously found egocentric interference effects could be replicated with German- (and Turkish) speaking adults using the paper-pencil version of the Sandbox task (e.g., Coburn et al., 2015). The first pilot study utilized simple materials displaying just a sandbox drawing on the paper and the location markers (as done by the previous studies, e.g., Coburn et al., 2015; Mahy et al., 2017). The second pilot study was very similar to the first one, except it used more engaging materials which displayed elaborate scenes. We conducted this second pilot study to control for our criticism of Study 1 (i.e., "We suspect that the not-so-engaging task materials might have caused our remaining participants to fail to pay enough attention to the task, which could have made the task less reliable.") The other difference between the first and the second pilot study was the task language (and participants' native language). As we did not find any difference between German- and Turkish-speaking participants in Study 1 and since Turkish-speaking participants were easier to reach at the time of the second pilot study, Pilot Study 2 tested Turkish-speaking participants.

Pilot Study 1

Method

Participants

Participants were recruited through personal communication channels and print ads. All participants were tested in person in a quiet room without any distractions. We used G*POWER (Faul et al., 2009) to conduct a power analysis and determine the sample size. We aimed to obtain .95 power to detect a medium effect size of .50 at the standard .05 alpha error probability with a more conservative two-tailed paired-samples t-test. The analysis revealed a required sample size of 54 participants. We tested 55 participants to achieve a sample size of 54 (one participant was excluded from the dataset as she was not a native German speaker and showed poor understanding of the task). Thus, the final sample consisted of 54 German-speaking adults: (24 females, 20 males, 10 unknown, M_{age} = 26.8, age range: 18 to 65). All participants provided written consent for the study and received a candy bar after the test session.

Materials (The Sandbox Task)

The scenarios used in our study were based on those used by Coburn et al. (2015) and Mahy et al. (2017). They always followed the same storyline: Agent A hides an object in Location 1, but then the object is transferred to Location 2 by Agent B in the absence of Agent A. The stories were always presented with accompanying images. The images (29.5 x 21 cm) displayed a rectangular container (21.9 x 2.7 cm) positioned in the middle of the image and text above the container. The crosses (0.5 x 0.5 cm) on the container indicated a hidden object's initial and final locations. These locations were always 13.4 cm apart, but their relative position changed across trials to prevent participants from learning the locations. In all of our studies, the direction of relocation was counterbalanced: in half of the trials, the object was transferred from left to right, and in the other half, the transfer was from right to left. The objects always crossed the midline of the sandbox during the transposition.

Once the scenarios were presented, participants worked on a word-search puzzle for 20 seconds. Puzzles prevented using perceptual cues to answer the question and were created by inserting family-related words into a 21 x 21 word-search puzzle using a puzzle maker website (https://puzzlemaker.discoveryeducation.com).

After the distraction task, participants were asked either where Agent A, who had a false belief about the object's location, would look for the object upon return (experimental trials; "Where will X look for the object?") or where s/he hid the object before leaving the scene (control trials; "Where did X hide the object?"). In both of these trials, the correct answer was around Location 1. Participants were expected to deviate in the direction of Location 2 in the experimental trials as they knew that the object was actually at Location 2, and this knowledge was expected to interfere with their judgments of others' perspectives and behaviors.

Design & Procedure

All participants completed the paper-pencil version of the Sandbox task, which aimed to tap egocentric biases. Each participant completed four experimental and four control trials presented in blocks (the order of the blocks counterbalanced) and one filler trial in between.

After consenting to the study, participants were seated at a table along with the experimenter. The experimenter was responsible for reading the stories and question prompts out loud, and she moderated the test session (i.e., proceeded across trials, made sure that participants saw only one location at a time, and managed the timing for distraction task).

After each scenario, there was a 20-seconds of puzzle solving, and then participants were presented with the question and asked to mark their answers on an empty sandbox drawing. The study took approximately 10 minutes.

Bias Calculation & Analyses

Biases were inferred from the object location measure: the horizontal distance (in cm) between the correct location (i.e., L1) and the participant's response. If the participants' responses were biased toward the wrong location (i.e., between the right and wrong answer, toward to middle of the paper), they received a positive object location value. The responses biased away from the wrong location (i.e., toward the edge of the Sandbox/paper rather than the middle) received a negative object location value. Once the object location measure was computed for each trial, we calculated the average object location measure in experimental and control trials for each participant. The averages were calculated in two ways: a) all responses were included in the averages (as done in the original Sandbox task studies), and b) the completely wrong answers (i.e., responses that were closer to the incorrect location than the correct location) were excluded from the averages. The latter method aimed to exclude the trials to which participants did not pay enough attention. We argue that adults are expected to have full-fledged perspective-taking abilities; therefore, completely wrong answers would reflect participants' failures of attention and could be excluded from the data for explorative purposes (e.g., does a bias exist when only the attended trials are considered?). The average scores were then used to deduce biases: if the average deviation in the experimental trials is bigger than the control trials, this indicates bias. As a result, different within-subject comparisons (paired-sample t-tests) were conducted with and without wrong answers to see if a bias exists in different conditions and groups. We used non-parametric tests (e.g., matched-pair Wilcoxon signed-rank Test) when the response data were not normally distributed. Even when non-parametric tests were more appropriate due to the non-normal distribution of the data, we also ran parametric tests as we had initially expected a continuous distribution of answers. The pattern of results and significance remained the same across all analyses.

Results

We first compared the average biases in control versus experimental trials without the wrong answers. No difference was detected between experimental (M=-1.40 SD=.89) and control (M=-1.47 SD=.83) trials, $t(53)$=.906, p=.369, d=.12. Then we included the wrong

answers in the data and repeated the comparisons. Again, no difference was revealed between experimental (*M*=.49 *SD*=2.82, *Mdn*=-.59) and control (*M*=-.27 *SD*=2.24, *Mdn*=-1.11) trials, $Z = -1.520$, $p = .129$.

Pilot Study 2

Method

Participants

Participants were recruited through personal communication channels and e-mail announcements. All participants were tested in person in a quiet room without any distractions. The sample size was based on the same rationale as the first pilot study; 54 participants were needed. We tested 56 participants to achieve a sample size of 54 (one participant was excluded from the dataset as she was not a native Turkish speaker, and one participant could not answer all trials as the test session was interrupted). The final sample consisted of 54 Turkish-speaking adults: (23 females, 31 males, $M_{age} = 25.6$, age range: 21 to 67). All participants provided written consent for the study and received a candy bar after the test session.

Materials (The Sandbox Task)

The materials used in the second pilot study were different from the first one in terms of the visual materials. Instead of showing dull materials with only a box drawing and the location markers, more engaging and elaborative materials have been displayed to the participants in the current study. For example, story-compatible background images were added; the object locations were not marked with Xs, but drawings of objects were shown; agents were displayed on the materials; and colorful materials were used. The images (29.5 x 21 cm) used in this study displayed a continuous rectangular area (width: 28.8 cm) positioned in the middle of the image and text at the bottom of the image. A different object was displayed for each trial; however, their surface area was kept constant across trials (2.5 cm^2). Objects' initial and final locations were always 20.3 cm apart. Besides these differences, the materials used in the current study were the same as the first pilot study.

Design & Procedure, and Bias Calculation & Analyses: same as Pilot Study 1.

Results

We first compared the average biases in control versus experimental trials without the wrong answers. No difference was detected between experimental (*M*=1.19 *SD*=.74) and control (*M*=1.2 *SD*=.77) trials, *t*(53)=-.070, *p*=.945, *d*=-.009. Then we included the wrong answers in the data and repeated the comparisons. Again, no difference was revealed between experimental (*M*=1.56 *SD*=1.81, *Mdn*=1.23) and control (*M*=1.45 *SD*=1.29, *Mdn*=1.27) trials, *Z* = -.065, *p* = .949.

Self-Construal Scale (Singelis, 1994)

Example Items:

**Item**	**1**	**2**	**3**	**4**	**5**	**6**	**7**
I enjoy being unique and different from others in many respects.	Strongly Disagree	Disagree	Somewhat Disagree	Don't Agree or Disagree	Somewhat Agree	Agree	Strongly Agree
I can talk openly with a person who I meet for the first time, even when this person is much older than I am.	Strongly Disagree	Disagree	Somewhat Disagree	Don't Agree or Disagree	Somewhat Agree	Agree	Strongly Agree
Even when I strongly disagree with group members, I avoid an argument.	Strongly Disagree	Disagree	Somewhat Disagree	Don't Agree or Disagree	Somewhat Agree	Agree	Strongly Agree
I have respect for the authority figures with whom I interact.	Strongly Disagree	Disagree	Somewhat Disagree	Don't Agree or Disagree	Somewhat Agree	Agree	Strongly Agree

Additional Analyses

Response Time Measure

Following a reviewer's suggestion, we conducted some additional analyses with the response time data. Using this measure, we identified and excluded the too quick and too slow outliers and repeated the within-subject analyses reported in the paper. Furthermore, we also used this data to compare the control and experimental trials in terms of response times.

Calculation of Response Time Measure

Response time measure was not one of the measures in which we were interested from the beginning. Therefore, we do not have direct measures for response times in our studies. In Study 1, we only have the information about the total duration of the study, which is not very informative for the analyses in this section. Thus, Study 1 will not be included in the investigations done with response times.

In Studies 2 & 3, we did not measure the response time directly. As an indirect measure, we can use the time series data from the mouse-tracking measure. It is, however, important to point out that this data would give us only the time that was taken to move the mouse around but not the overall time spent on a trial. Therefore, its validity as a response time measure is unclear.

Within-subject comparisons without the response time outliers

The response time data in Study 2 revealed four very slow outliers in the altercentric bias condition (one from the English-speaking sample and three from the German-speaking sample) and three very slow outliers in the egocentric bias condition (all from the English-speaking sample). We removed these outliers and repeated the within-subject comparisons with the object location and mouse-tracking measures. The overall result pattern did not change: neither an altercentric nor an egocentric bias was found in any group. More specifically, no difference between experimental and control trials was observed for English-speaking participants with object-location measure, neither in egocentric ($Z = -1.723$, $p = .085$) nor altercentric ($Z = -1.069$, $p = .285$) bias condition. No difference was revealed by mouse-tracking measures, neither in egocentric ($Z = -1.425$, $p = .154$) nor altercentric ($Z = -.628$, $p = .530$) bias condition.

Similarly, no difference between experimental and control trials was observed for German-speaking participants in the altercentric bias condition, neither with object-location ($Z = -.467$, $p = .641$) nor with mouse-tracking ($Z = -.537$, $p = .591$) measure. As the response time data did not reveal any outliers for German-speakers in the egocentric bias condition, the analyses were not repeated for egocentric bias in this group.

Study 3 revealed only two slow outliers. When those were eliminated from the data, no change in the result pattern was observed: no difference between experimental and control trials was observed with object location measures, neither for egocentric (Z = -.403, p = .687) nor altercentric (Z = -1.336, p = .181) bias. Also, no difference between experimental and control trials was observed with mouse-tracking measures, neither for egocentric (Z = -1.678, p = .093) nor altercentric (Z = -.778, p = .437) bias.

Mixed-Models

Following the suggestion of one reviewer, we used a mixed-effects model approach to see if the individual bias scores are influenced by the trial type (i.e., experimental and control). More specifically, we conducted mixed-effect models for individual bias scores with the trial type as the fixed effect and the participant and item as the random effects.

In Study 1, the trial type had no effect on the bias score for German (egocentric bias: F=.87, p = .81, 95% CI [-81.34, 98.96]; altercentric bias: F=.46, p = .524, 95% CI [-91.24, 160.98]) and Turkish participants (egocentric bias: F=4.49, p = .08, 95% CI [-163.33, 11.72]; altercentric bias: F=.37, p = .565, 95% CI [-169.87, 102.11]).

In Study 2, we again found no effect of trial type on bias scores as measured by the object location measure, neither for German- (egocentric bias: F=.49, p = .513, 95% CI [-20.65, 11.64]; altercentric bias: F=.04, p = .852, 95% CI [-34.68, 29.53]) nor English-speaking (egocentric bias: F=1.60, p = .209, 95% CI [-12.71, 57.43]; altercentric bias: F=.16, p = .704, 95% CI [-31.04, 42.26]) participants. Mouse-tracking measures revealed similar results for both German- (egocentric bias: F=.07, p = .792, 95% CI [-5.99, 4.57]; altercentric bias: F=.74, p = .390, 95% CI [-6.41, 2.51]) and English-speaking (egocentric bias: F=.33, p = .568, 95% CI [-3.69, 6.70]; altercentric bias: F=.00, p = .990, 95% CI [-6.90, 6.83]) participants.

Study 3 provided similar line of results. Namely, no effect of trial type was observed regardless of the measure, i.e., object location (egocentric bias: F=.00, p = .955, 95% CI [-17.215, 17.98]; altercentric bias: F=1.73, p = .190, 95% CI [-2.53, 12.68]) or mouse-tracking (egocentric bias: F=1.06, p = .374, 95% CI [-10.13, 5.04]; altercentric bias: F=1.33, p = .321, 95% CI [-5.57, 12.75]).
